# Delivery mode-associated gut microbiota in the first 3 months of life in a country with high obesity rates: A descriptive study

**DOI:** 10.1097/MD.0000000000022442

**Published:** 2020-10-02

**Authors:** Chiharu Murata, Pedro Gutiérrez-Castrellón, Fernando Pérez-Villatoro, Itzhel García-Torres, Sergio Enríquez-Flores, Ignacio de la Mora-de la Mora, Cynthia Fernández-Lainez, Julieta Werner, Gabriel López-Velázquez

**Affiliations:** a Research Methodology Department. National Institute of Pediatrics, Mexico; b General Hospital Dr. Manuel Gea González; c Winter Genomics; d Group of Research on Biomolecules and Infant Health, IEM&S Lab. INP, México; e Laboratory of Inborn Errors of Metabolism and Screening, INP, and Postgraduate in Biological Sciences, UNAM, México. Actual Address: Immunoendocrinology, Division of Medical Biology, Department of Pathology and Medical Biology, University of Groningen and University Medical Center Groningen, Groningen, The Netherlands; f Indigenous Services Canada, Thunder Bay, Ontario, Canada.

**Keywords:** microbial profiles, proteobacteria, newborns

## Abstract

Delivery methods during childbirth and their related gut microbiota profiles have important impacts on health later in life, they can contribute to the development of diseases such as obesity, whose highest prevalence rate is found among the Mexican child population. Coincidentally, Mexico has one of the highest global average annual rate increase in cesarean births (C-section). Since Mexico leads the world in childhood obesity, studying the relationship between childbirth delivery methods and gut microbiota profiles in this vulnerable population may be used to identify early risk factors for obesity in other developed and developing countries. The objective of this study is to determine the association between child delivery method and gut microbiota profiles in healthy Mexican newborns.

Fecal samples of 57 term infants who participated in a randomized clinical trial in 2013 to study the safety of *Agave* fructans in newborns, were used in this study. DNA samples were extracted and used to characterize the microbiota composition using high-throughput 16S rRNA gene sequencing. The samples were further divided based on childbirth delivery method, as well as early diet. Gut microbiota profiles were determined and analyzed using cluster analysis followed by multiple correspondence analysis.

An unusual high abundance of Proteobacteria was found in the gut microbiota of all Mexican infants studied, regardless of delivery method. Feces from infants born by C-section had low levels of Bacteroidetes, high levels of Firmicutes, especially *Clostridium* and *Enterococcus*, and a strikingly high ratio of Firmicutes/Bacteroidetes (F:B). Profiles enriched in Bacteroidetes and low F:B ratios, were strongly associated with vaginal delivery.

The profile of gut microbiota associated with feces from Mexican infants born by C-section, may be added to the list of boosting factors for the worrying obesity epidemic in Mexico.

## 1 Introduction

Several factors may synergize to promote the complex mosaic of health outcomes that characterize populations with high rates of chronic diseases. The World Health Organization (WHO) recommends rates of C-sections births to be below 15%, but many countries far exceed such recommendation. North American countries show rates as high as 32.3% in USA and Canada, and 32.8% in Mexico.[^1^,^2^,^3^] The USA and Canada have the lowest global average annual rate increase in cesarean births (1.6%), while Mexico has one of the highest (∼4.1%).^[3]^ This stark contrast should be understood and prevented by providing educational strategies to clinicians and patients regarding the benefits of vaginal birth,^[4]^ and the risk of unnecessary C-sections.[^5^,^6^] However, scientific evidence is needed to support this decision-making process.[^7^,^8^,^9^]

Canada is one of the countries with more studies addressing unnecessary C-sections,[^10^,^11^,^12^,^13^] however the rate has not decreased enough (27.9% in 2015^[14]^) to meet the recommendations of the WHO. The inability to link maternal and neonatal health records results in missing information needed to efficiently prevent C-sections.[^5^,^15^]

Delivery method alters infant's gut microbiota, resulting in the development of diseases such as obesity, type 1 diabetes, asthma, allergies, and even neurodevelopmental disorders.[^16^,^17^,^18^] Obesity is the other face of malnutrition and is the most blatantly visible – yet the most neglected – public health problem. Mexican infants are exposed to an obesogenic environment, with problems often seen in developing countries (eg, vitamin D and iron deficiencies), but also those often seen in developed countries (eg, obesity).[^19^,^20^]

Our study focuses on this topic because Mexico leads the world in childhood obesity.^[20]^ Gut microbiota is considered a new target for interventions aiming to close the vicious cycle of obesity.^[21]^ Therefore, the consequences of decisions regarding childbirth delivery methods and their possible role as a risk factor are not to be underestimated. Finding differences in gut microbiota patterns in Mexican infants delivered by C-section or vaginal birth could be of help in dictating new policies in public health. In the current study we used high-throughput 16S rRNA gene sequencing to analyze infant gut microbiota,[^22^,^23^,^24^,^25^,^26^] to identify the association between delivery method and gut microbiota profiles of healthy Mexican newborns.

## 2 Methods

### 2.1 Study design

This is a descriptive study based on the analyses of stool samples, obtained from a subsample of newborns participating in clinical trial (*ClinicalTrials.gov* identifier NCT01251783). We performed a convenience sampling from the available stool samples (57 samples). In the original study, stool samples were collected from healthy Mexican term infants (38.4 ± 3.6 weeks of gestation) whose mothers were recruited at Mexico City's Instituto Nacional de Pediatria (INP) for a prospective, double blind, randomized controlled trial study to study the safety and efficacy of prebiotic *Agave* fructans when added to infant formula. The study was conducted from February to August 2010.^[27]^ Infant stool samples were collected at birth (12 ± 4 days) and at 3 to 4 months after birth for analysis of gut microbiota.

Infants in the study were fed by:

(1) exclusively breastfeeding,
(2) infant formula added with prebiotics and probiotics, or
(3) exclusively infant formula.

All mothers gave their informed consent for inclusion in the study before any sample was collected. This study was approved by the Health Research Ethics Board of the INP (Registry: 076/2009). The present work is a cross-sectional study.

### 2.2 Stool sample analysis and sequencing

To avoid cross-contamination by the parents during stool handling, chemical-free diapers were used to collect samples at the time of pediatric appointments at the INP, México. Collected samples were chilled on ice and taken to the laboratory to isolate bacterial DNA using a QIAamp DNA stool mini kit (Qiagen) as described.^[28]^ DNA samples were eluted in a final volume of 100 μl of water and used for high-throughput signature gene sequencing to identify individual organisms. The variable regions V1-V3 of 16S rRNA were used as signature gene, which can be used to identify individual organisms.^[29]^ Three libraries were generated to sequence approximately 10,000 *reads* per sample. A Roche 454-GS FLX Titanium sequencing system was used.

### 2.3 Analysis of bacterial diversity

The obtained sequences were multiple aligned to generate a pairwise distance matrix with the PyNAST and UCLUST packages. The RDP program was used to assign the genus, and RITA program to assign the species.^[30]^ Taxonomic assignments were compared with databases for rRNA. The biodiversity measures of Shannon index and Chao 1 score were calculated for each individual at birth and at 3.5 months of age using QIIME software package (http://qiime.org/). Bacterial relative abundances are reported with median and interquartile range (IQR) at 3 taxa levels, which include phylum, family, and genus.

### 2.4 Statistical analysis

Relative abundances were compared with the variable “delivery method” by Student *t* test for independent samples. To determine the association of bacterial relative abundance with the delivery method, cluster analysis was performed to obtain groups with different bacterial distribution patterns at phylum level, followed by multiple correspondence analysis (MCA). The clusters were obtained by hierarchical clustering with Ward's method,^[31]^ the number of clusters were determined based on the cubic clustering criterion (CCC). MCA included three variables: cluster of bacterial distribution pattern; birth method and age. Throughout the study, *P* < .05 was considered statistically significant. Descriptive statistics mean comparison and cluster analysis were performed by JMP11 (SAS Institute, Inc) and MCA was performed using FactoMineR^[32]^ and Factoextra^[33]^ within R environment.

## 3 Results

### 3.1 Study population

Stool samples were collected from 57 healthy term infants; samples were collected at birth and at 3.5 months of age. Mean age at birth was 12.19 ± 3.88 days, and mean age at the second sampling was 3.5 months ± 6.2 days. The study population included 31 girls (54%) and 26 boys (46%); 29 infants (50.8%) were born by cesarean section, 17 girls (29.8%) and 12 boys (21%) (Table 1). Fourteen infants were exclusively breastfed (24.56%) and 43 were not breastfed (75.44%), 17 were fed with conventional infant formula (30.35%), and 26 with infant formula enriched with probiotics and prebiotics (*Agave* fructans) (44.64%) (Table 1). Breastfeeding only was almost twice more common among infants delivered vaginally compared to C-section (15.78% *vs* 8.77%, respectively). No infants received antibiotics during the study.

**Table 1:**
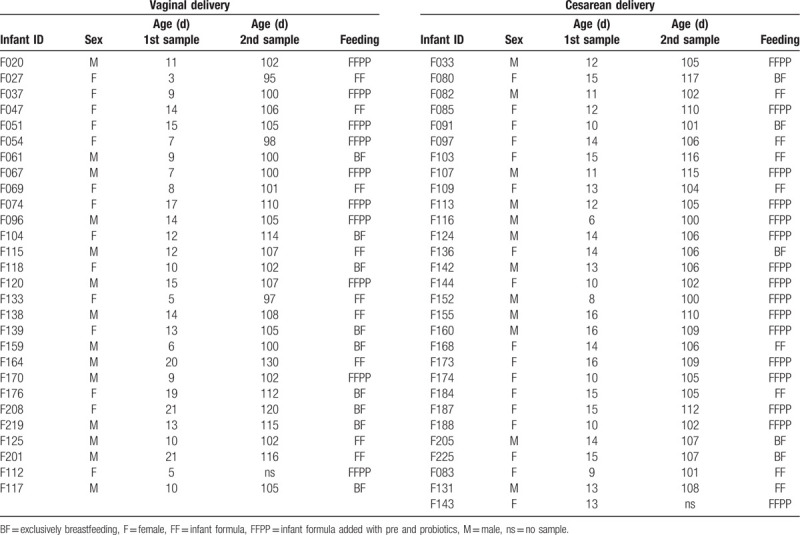
Characteristics of infants in study population.

### 3.2 Relative abundance and profiles of gut microbiota

**Figure 1:**
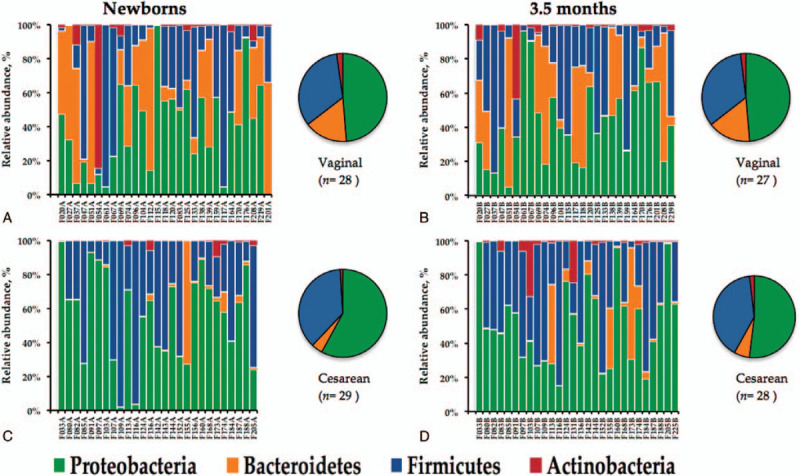
Composition of microbiota from fecal samples of 57 healthy infants at the phylum level. Vaginal delivery at birth (A), and at 3.5 months of age (B), and cesarean section at birth (C) and at 3.5 months of age (D).

Median values of dominant phyla, families and genera in the infants at birth and 3.5 months of age are presented in Table 2. Fecal microbiota profiles, both at birth and at 3.5 months of age were generally dominated by the phylum Proteobacteria (49.8% and 45.7%, respectively) with representation mainly by the genera *Enterobacter*-*Escherichia*-*Klebsiella*. The second most abundant phylum was Firmicutes (mean of 25.9%, and 29.9%, respectively) with diverse representation from several genera. The less abundant phylum was Actinobacteria (mean 0.41%). As for other populations, high variability of microbial abundance was found between individuals (Fig. 1). Nonetheless, significant differences were found between the abundance of several taxa and the delivery method (Table 3). At birth, the abundance of the genus *Bifidobacterium* was higher (almost 7-fold) in infants delivered vaginally (*P* .323). Compared to infants delivered vaginally, those born by C-section had significantly lower Bacteroidetes communities both at birth and at 3.5 months of age (*P* < .001, *P* = .010, respectively). This was observed especially for the *Bacteroides* genus (see Table 3). For infants born by C-section, the abundance of the genus *Streptococcus* was significantly lower at birth (*P* .030). On the other hand, the phylum Proteobacteria and genera *Enterobacter*-*Escherichia*-*Klebsiella*, *Clostridium*, and *Enterococcus* were significantly higher at birth in infants born by C-section (see Table 3).

**Table 2:**
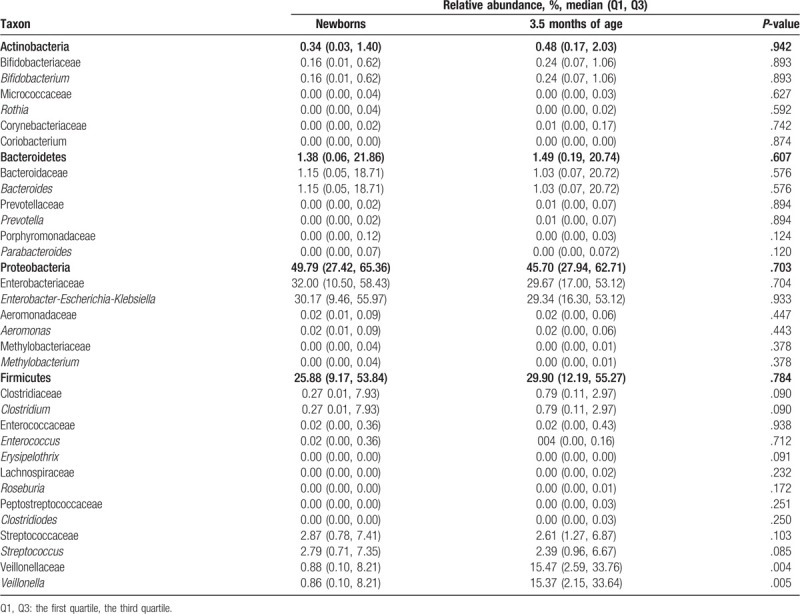
Relative abundance and frequency of dominant phyla, families and genera in fecal samples at birth and 3.5 months of age.

**Table 3:**
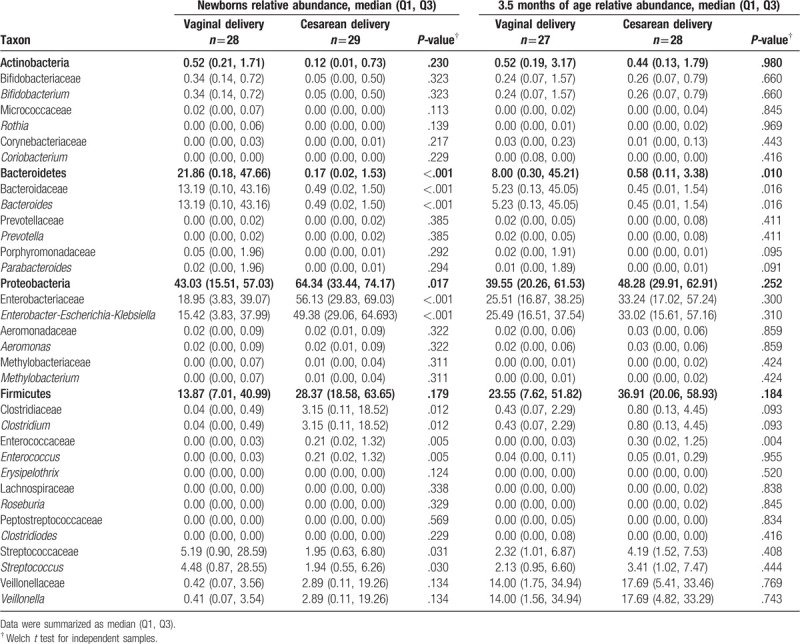
Relative abundance and frequency of dominant phyla, families, and genera in fecal samples by mode of delivery at birth and 3.5 months of age.

Infants were fed with breast milk (BF) or 5 different combinations of prebiotics and probiotics in infant formula (see Table 1). Stratified comparisons of delivery method by infant diet were not conducted as sample sizes would have decreased to ∼4 individuals per group. Nonetheless, we analyzed the data from infants grouped in those fed with prebiotics and probiotics (FFPP) comparing with those BF or fed with infant formula (FF). Results were not of statistical significance, but we found that bacterial abundances among some groups of feeding tend to show differences (at 3.5 months of age). Table 4 shows a trend to higher abundance of Actinobacteria (especially *Bifidobacterium* genus) and Firmicutes in FF group compared with BF and FFPP. Conversely, Bacteroidetes showed a trend to lower abundance between FF vs BF and FFPP. These bacterial groups were closer in abundance between infants of BF and FFPP. Abundance of *Clostridium* and *Enterococcus* was lower only in those infants breast-fed (Table 4).

**Table 4:**
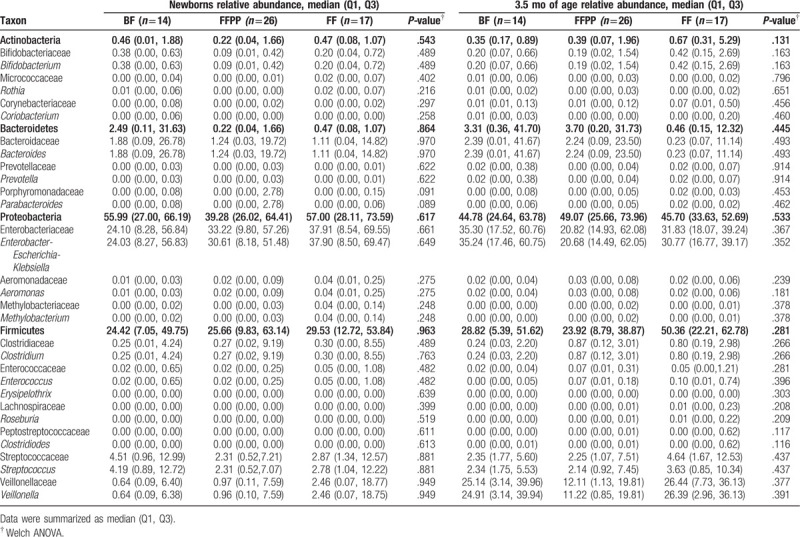
Relative abundance of dominant phyla, families, and genera in fecal samples by feeding at birth and 3.5 months of age.

### 3.3 Firmicutes/Bacteroidetes ratio

The medians of Firmicutes/Bacteroidetes ratios (F:B ratio) were 0.63 and 2.9 in infants born vaginally at birth and 3.5 months of age, respectively. Infants born by C-section showed F:B ratios of 167 and 63.6 at birth and 3.5 months of age, respectively. When grouped by feeding allocation, the medians of F:B ratio were as follows at birth: BF: 9.8, FFPP: 116.6, and FF: 62.82. After 3.5 months, the medians of F:B ratios were: BF: 8.7, FFPP: 6.46, and FF: 109.47.

### 3.4 Association of gut microbiota with delivery method and age

**Figure 2:**
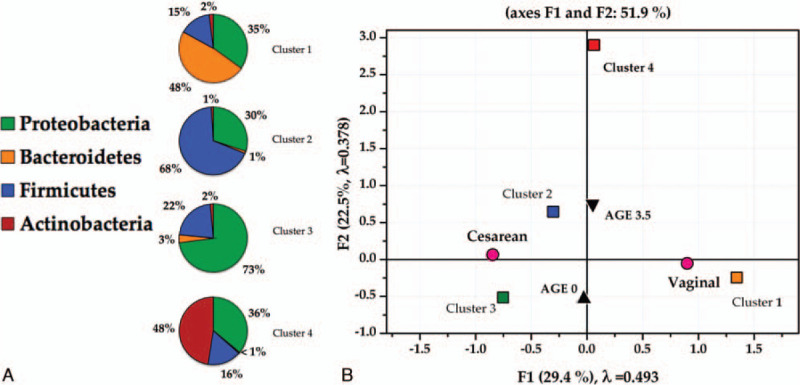
Association with delivery method and age of gut microbiota profiles from fecal samples. Four clusters of infants were identified by means of hierarchical clustering according to bacterial distribution patterns (A) and their distribution after multiple correspondence analysis according to delivery method and age (B).

After hierarchical clustering, we identified 4 clusters of infants according to the different gut bacterial distribution patterns for each individual (Fig. 2A). The F:B ratios were 0.31, 68, 6.3, and 32 for clusters 1 to 4, respectively. When MCA was applied to the microbial relative abundance and delivery method, we identified a differential distribution of phyla according to delivery method (Fig. 2B). Since the F1-axis of the symmetric plot of MCA explains 29.7% of the relationship variables, the strongest positive association was found between vaginal delivery and cluster 1 (highest abundance of Bacteroidetes and the lowest F:B ratio), whereas cluster 3 shows the strongest negative association with vaginal delivery (Fig. 2B). Cesarean delivery is associated, firstly with cluster 3 (the highest abundance of Proteobacteria), and secondly with cluster 2 (the highest abundance of Firmicutes and the highest F:B ratio). Cluster 4 is the only associated with the infants at 3.5 months of age (highest abundance of Actinobacteria).

### 3.5 Microbial richness and diversity

The mean rarefied Chao 1 score for species richness of fecal samples was 126.83 (range 24–328.94) at birth, and 146.64 (range 25–383.9) at 3.5 months old. The mean Shannon diversity index was 2.37 (range 0.63–3.67) at birth, and 2.76 (range 0.48–4.30) at 3.5 months old. Neither age, nor delivery method or sex showed significant differences in richness and diversity between groups (Table 5).

**Table 5:**
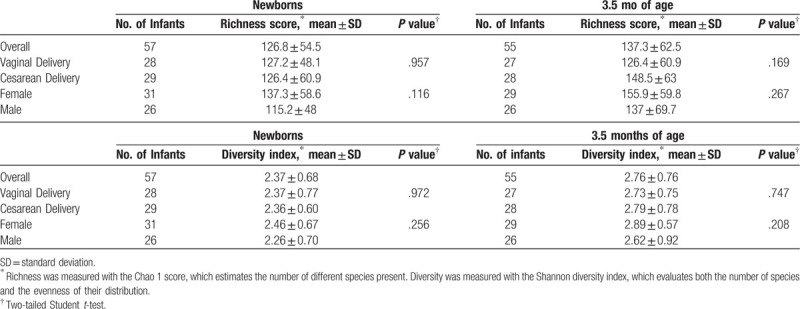
Richness and diversity of fecal microbiota in infants, by age, delivery mode, and sex.

### 3.6 Disease burden

In Mexico, disease burden caused by the three most important diseases related to obesity affected 90.74 million people, representing 72.7% of the total population in the country in 2017 (https://vizhub.healthdata.org/gbd-compare/). Table 6 shows disease burden by measuring years lived with disability, disability adjusted life year, and deaths.

**Table 6:**
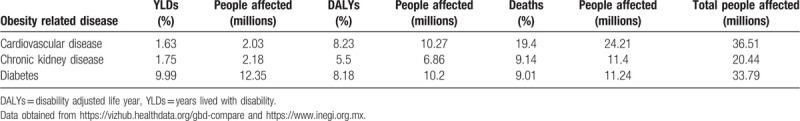
Total disease burden in Mexico measured in YLDs, DALYs, and deaths by the three most related obesity diseases.

## 4 Discussion

Obesity is associated with increases in annual health-care costs of 36% and medication costs of 77% compared with being of average weight.^[34]^ Also, is associated with long-term negative economic consequences. Children with obesity were absent from school significantly more (12.2 ± 11.7 days) than children who were considered to be of normal weight (10.1 ± 10.5 days). Obesity was associated with 1.9 more days absent after controlling for age, gender, race/ethnicity, and school.^[35]^ In addition, children who are obese or overweight are at increased risk for being the target of aggressive behavior from their peers.^[36]^ If obesity could be addressed early in life, it could have a substantial impact on healthcare costs. It is estimated that if the number of individuals ages 16 and 17 who are overweight or obese could be reduced by 1%, then the number of adults with obesity in the future could be reduced by 52,812; this would result in a decrease in life-time medical costs of $586 million dollars.^[37]^

In adults and infants Firmicutes and Bacteroidetes dominate the gut microbial community.^[38]^ We found the phylum Proteobacteria as the most abundant in Mexican infants participating in this study, regardless of the delivery method and age. Proteobacteria in this population far exceeds the abundance of any other bacterial population. At birth, abundance of Proteobacteria was higher in those infants born by C-section compared to those born by vaginal delivery. C-section was strongly associated with the cluster showing the highest abundance in Proteobacteria (Fig. 2, Table 3). Also, at 3.5 months of age this phylum is the most abundant regardless of delivery method but still higher in infants born by C-section (Table 3).

These findings suggest that an increased prevalence of Proteobacteria in infants older than 3.5 months old could be possible used as a marker for unstable microbial communities. The amount of these bacteria could be of help to suspect a risk of developing diseases like obesity during adulthood.^[39]^ The abundance of this group of microbes in infants could be a reflection of the mother's gut microbiota since it is known that the proportion of Proteobacteria in the gut of pregnant women increases during the later period of pregnancy.^[40]^ In fact, Proteobacteria can be transferred from the maternal placenta through fetal swallowing of amniotic fluid in utero.^[39]^

The high abundance of Proteobacteria in Mexican infants could represent a risk factor to develop diseases in the future or possible play a role in preparing the gut for successive colonization by strict anaerobes. The enrichment of Proteobacteria found in this infant population is higher than that found directly in soil,[^39^,^41^] and remained since birth until 3.5 months of age.

Scientific evidence supporting our claim comes from studies as those reported by Fei and Zhao, where they found an increase in the Enterobacteriaceae family (included in the phylum Proteobacteria) in an obese volunteer.^[42]^ The Enterobacteriaceae was also the most abundant family of Proteobacteria reported in the sample of our study (see Table 2). Additionally, it is reported that after weight loss, the Enterobacteriaceae population is the most affected, with a significant reduction in abundance. Moreover, germfree mice inoculated with a strain of *Enterobacter* isolated from the volunteer's gut, induced fully developed obesity and insulin resistance on a high fat diet but not on normal chow diet, whereas the germfree control mice on a high-fat diet did not exhibit the same disease phenotypes.^[42]^ Also, one of the most abundant genera of Proteobacteria found in our study was *Enterobacter* (see Table 2).

Analysis of microbiota composition in children has demonstrated a gradual increase in Proteobacteria among healthy, obese, and nonalcoholic steatohepatitis children (nonalcoholic steatohepatitis is a serious liver disease associated with obesity).^[43]^ When analyzing at family and genus levels, it was found that this difference was sustained by an increase in Enterobacteriaceae and *Escherichia*, respectively; again, a family and a genus abundantly found in our study (Table 2).

On the other hand, some authors hypothesize that gut microbiota may induce alterations in the gut-brain axis to explain its role in metabolic diseases. On this line, Vaughn et al found that rats fed with high-fat diet were associated not only with microbiota variations, in particular with proliferation of Proteobacteria, but also with reorganization of vagal afferents and microglia activation in the nucleus of the solitary tract, the brain center that modulates satiety.^[44]^

The rationale to state that gut Proteobacteria could be linked to obesity development is the following. A common trait of Proteobacteria is the presence of lipopolysaccharide in the outer membrane.^[45]^ A connection between low-grade inflammation, sustained by lipopolysaccharides, and the development of metabolic disorders is well established.^[46]^ In fact, lipopolysaccharide endotoxin is the only known bacterial product which, when subcutaneously infused into mice in its purified form, can induce obesity and insulin resistance via an inflammation mediated pathway.^[42]^ Besides, epidemiological studies show increased population of lipopolysaccharide producers and elevated lipopolysaccharide load in various obese cohorts.[^47^,^48^] Additionally, Cani et al demonstrated that metabolic concentrations of plasma lipopolysaccharides are a sufficient molecular mechanism for triggering insulin resistance, obesity and type 2 diabetes.^[49]^ Notably, inflammation is demonstrated to be implicated in the development of metabolic disorders, such as obesity, diabetes, and nonalcoholic fatty liver disease/nonalcoholic steatohepatitis. Many studies on these topics are based on the comparison of microbiota composition in health and disease with frequent observation of increased abundance of Proteobacteria in the latter group.^[45]^ A possible mechanism that could allow the access of lipopolysaccharides produced by Proteobacteria in the bloodstream is the increase of intestinal permeability caused by reduction on the expression of genes coding for proteins of the tight junctions. Such condition was experimentally induced with high-fat feeding in mice.^[49]^

Also, we observed a drastic decrease in relative abundance of Bacteroidetes in infants born by C-section, this is similar to that reported for infants in developed countries like Canada^[11]^ and Sweden.^[50]^ Bacteroidetes generally make up half or more of the gut microbiome[^51^,^52^] but we found a very low abundance in infants born by C-section, whereas vaginally delivered infants showed relative abundance of Bacteroidetes to be near 30%. Low abundance of the phylum Bacteroidetes is associated with obesity in infants and with low circulating levels of Th1-associated chemokines, which diminishes the natural immune response.[^52^,^53^]

The F:B ratio was higher in infants born by C-section and the cluster with the lowest F:B ratio was strongly associated with vaginal delivery. The F:B ratios in those individuals born by C-section far exceed the values reported in any other infant populations.[^50^,^54^] The F:B ratio is regarded to be of significant relevance in human gut microbiota composition,^[55]^ and high ratios are associated with the development of diseases and obesity.[^39^,^53^,^54^,^55^,^56^,^57^] High values in F:B ratios have been reported in Canadian infants^[11]^; however, this condition has not being further studied. In infants born by C-section the genera *Enterococcus* and *Clostridium* were enriched, which has been associated with obesity in infants, adolescents, and adults.[^58^,^59^]

The differences in gut microbial communities found in this study, may be potential contributors to the well-known health condition of the Mexican children (and thereafter in adults). First, the outstanding and unusual high abundance of the phylum Proteobacteria in the whole infant population studied here. The high abundance of these microorganisms was observed in all infants independently of the delivery method and age. Second, influence of delivery method in the gut microbiota profiles. This study showed that those infants born by C-section had a significant decrease in the abundance of Bacteroidetes, enriched in *Enterococcus* and *Clostridium* genera, and strikingly high values of F:B ratio, factors associated in other populations to obesity. Third, in addition to the factors described in this study, other factors including the presence of the polymorphism Gln233Arg in the leptin receptor of the Mexican population and its association with hemodynamic and metabolic disturbances related to obesity,^[60]^ the increased triglyceride levels in blood and altered propionic and butyric acid concentrations in stool samples of overweight and obese Mexican children,^[61]^ and the rising obesogenic environment found in México,[^62^,^63^] need to be taken into account when developing policies to prevent chronic diseases such as obesity.

Finding the patterns related to microbiota profiles in this population, could be helpful when developing public health policies aimed to address the lifelong health outcomes of vulnerable populations.

The power of the data was insufficient to analyze the combination of variables between delivery methods and feeding. Differences in richness and diversity of gut microbial communities in vaginally versus C-section delivered infants are important characterizing factors; however, our results did not show significant differences between groups. The study of vaginal and gut microbiome from mothers of the infant population studied could provide valuable information on the correlation between maternal and infant gut microbial profiles; however, the study was not designed to analyze such data.

## 5 Conclusions

Our data show that the delivery method largely influences intestinal microbiota in Mexican infants, and that C- section is one more factor that, along with the interacting genotype^[61]^ and obesogenic environment,[^62^,^63^] may contribute to obesity and other pathologies in Mexican children, as has been found for respiratory infections identified in the first year of life in other populations.^[64]^ In light of the disease burden data presented, the possible risk factor that we identified could lead to an important potential chronic infantile and adult disease. We propose that health policies should be developed to encourage vaginal delivery in an attempt to decrease the risk of developing obesity and other pathologies worldwide.

## Author contributions

**Conceptualization:** Pedro Gutiérrez-Castrellón.

**Data curation:** Itzhel García-Torres, Ignacio de la Mora-de la Mora, Cynthia Fernández-Lainez, Julieta Werner.

**Formal analysis:** Chiharu Murata, Fernando Pérez-Villatoro, Sergio Enríquez-Flores, Julieta Werner.

**Investigation:** Itzhel García-Torres, Sergio Enríquez-Flores, Cynthia Fernández-Lainez.

**Methodology:** Pedro Gutiérrez-Castrellón, Fernando Pérez-Villatoro, Itzhel García-Torres, Sergio Enríquez-Flores, Ignacio de la Mora-de la Mora, Cynthia Fernández-Lainez.

**Supervision:** Pedro Gutiérrez-Castrellón.

**Writing – original draft:** Pedro Gutiérrez-Castrellón.

MSc ChM is the main author, and made the statistical analyses.

Dr GL-V, Dr PG-C are the corresponding authors, conceptualized, designed the study, interpreted the results, wrote the original draft of the manuscript, and approved the final manuscript as submitted.

BSc FP-V made the genomic libraries and the bioinformatics.

Dr IG-T, Dr IdM-dM, Dr SE-F, MSc CF-L, Dr JW coordinated the collection of samples and data, processed the samples, assembled the databases, curated the data, approved the final version to be published and agreed to be accountable for all aspects of the work.
